# Untangling adaptive functioning of PMM2-CDG across age and its impact on parental stress: a cross-sectional study

**DOI:** 10.1038/s41598-023-49518-y

**Published:** 2023-12-20

**Authors:** Florencia Epifani, Susana María Pujol Serra, Marta Llorens, Sol Balcells, Gregorio Nolasco, Mercè Bolasell, Sergio Aguilera-Albesa, Ramon Cancho Candela, José Luis Cuevas Cervera, Verónica García Sánchez, Oscar Garcia, María Concepción Miranda-Herrero, Pedro J. Moreno-Lozano, Bernabé Robles, Susana Roldán Aparicio, Ramón Velázquez Fragua, Mercedes Serrano

**Affiliations:** 1 Neuropediatric Department, Hospital Sant Joan de Déu, Barcelona, Spain; 2 Mental Health Department, Hospital Consorci Sanitari Parc Taulí, Sabadell, Spain; 3 Pediatric Mental Health Department, Hospital Sant Joan de Déu, Barcelona, Spain; 4 Department of Statistics, Institut de Recerca Sant Joan de Déu, Barcelona, Spain; 5 Department of Genetic and Molecular Medicine IPER, Hospital Sant Joan de Déu, Barcelona, Spain; 6 Department of Pediatrics, Hospital Universitario de Navarra, Pamplona, Spain; 7 Servicio de Pediatría, Hospital Universitario Rio Hortega, Universidad de Valladolid, Valladolid, Spain; 8 Pediatric Neurology, Rare and Metabolic Diseases, Hospital Universitario de Jaén, Jaén, Spain; 9 Neuropediatric Department, Hospital Universitario Puerto Real, Cadiz, Spain; 10 Department of Pediatrics, Hospital Universitario Virgen de la Salud, Toledo, Spain; 11 Pediatric Neurology, Hospital General Universitario Gregorio Marañon, Madrid, Spain; 12 Internal Medicine Department, Muscular and Inherited Metabolic Disorders Adults Unit, Hospital Clínic de Barcelona, Barcelona, Spain; 13 Neurology Department, Hospital de Sant Boi, Parc Sanitari Sant Joan de Déu, Sant Boi, Spain; 14 Neuropediatric Department, Hospital Virgen de las Nieves, Granada, Spain; 15 Neuropediatric Department, Hospital Universitario La Paz, Madrid, Spain; 16 U-703 Centre for Biomedical Research On Rare Diseases (CIBER-ER), Instituto de Salud Carlos III, Passeig Sant Joan de Déu, 2, Esplugues, 08950 Barcelona, Spain

**Keywords:** Neurodevelopmental disorders, Neurodevelopmental disorders

## Abstract

Phosphomannomutase deficiency (PMM2-CDG) leads to cerebellar atrophy with ataxia, dysmetria, and intellectual deficits. Despite advances in therapy, the cognitive and adaptive profile remains unknown. Our study explores the adaptive profile of 37 PMM2-CDG patients, examining its association with parental stress and medical characteristics. Assessment tools included ICARS for the cerebellar syndrome and NPCRS for global disease severity. Behavioral and adaptive evaluation consisted of the Vineland Adaptive Behavior Scale and the Health of the Nation Outcome Scales. Psychopathological screening involved the Child Behavior Checklist and the Symptom Check-List-90-R. Parental stress was evaluated using Parental Stress Index. Results were correlated with clinical features. No significant age or sex differences were found. ‘Daily living skills’ were notably affected. Patients severely affected exhibited lower adaptive skill values, as did those with lipodystrophy and inverted nipples. Greater severity in motor cerebellar syndrome, behavioral disturbances and the presence of comorbidities such as hyperactivity, autistic features and moderate-to-severe intellectual disability correlated with greater parental stress. Our study found no decline in adaptive abilities. We provide tools to assess adaptive deficits in PMM2-CDG patients, emphasizing the importance of addressing communication, daily living skills, and autonomy, and their impact on parental stress in clinical monitoring and future therapies.

## Introduction

Congenital disorders of glycosylation (CDG) comprise some 160 genetic disorders caused by impaired synthesis of glycoconjugates, with phosphomannomutase deficiency (PMM2-CDG, *601785OMIM) being the most frequent, with more than 1,000 reported patients^1^. Early signs of PMM2-CDG include abnormal fat distribution, inverted nipples, strabismus, and hypotonia. Infants soon develop ataxia and psychomotor delay, but also show early multi-organic manifestations, such as failure to thrive, enteropathy, hepatic dysfunction, abnormal coagulation, and cardiac involvement^2–4^.

Cerebellar involvement is a major clinical feature of PMM2-CDG. At birth, most affected children have cerebellar hypoplasia, followed by progression towards atrophy^5,6^. Key neurological symptoms observed in patients, such as ataxia, dysmetria, tremor, abnormal eye movements, dysarthria, and cognitive deficits, may be attributed to the cerebellar syndrome. These symptoms significantly contribute to major disability in affected individuals. However, there is a lack of neuropsychological studies describing the psychological and adaptive functioning phenotypes or attempting to determine whether cognitive and functional deficits are due to infratentorial or supratentorial structures dysfunction.

The traditional view of cerebellum's involvement primarily in motor-related processes has evolved in recent decades. It is now widely recognized that the cerebellum plays a crucial role in cognition, including executive functions, processing speed, language, and various processes related to emotion-related disorders leading to the term ‘dysmetria of though’, to describe the deficits in higher-order behavior explained by the lack of cerebellar modulation^7–9^.

To date, there have been reports of large cohorts of PMM2-CDG patients, some of them reporting the cognitive issues related to PMM2-CDG^3,4,10^. However, there is limited research and description of the adaptive functioning and behavioral features of PMM2-CDG, despite existing evidence of the difficulties, observed by families and clinicians, and the urgent need due to the promising therapies that are developing and the clinical trials that are ongoing^11^. Furthermore, recent findings highlight the importance of addressing neurological and mental health issues from the perspective of PMM2-CDG patients and their caregivers^12^, as the condition greatly impacts their quality of life.

Parents of children with disabilities often report higher levels of parental stress, along with symptoms such as anxiety and depression^13^, related to their own coping strategies and the child's behavior and emotional difficulties^14^. Recent literature on parental stress in the context of rare genetic conditions emphasizes the importance of assessing more than just the intellectual functioning of individuals with these conditions^15^, attending to factors such as internalizing and externalizing symptoms, adaptive behavior, and physical health. In addition, the overall functioning of the parents and family needs to be considered. The caregivers physical, psychological and mental burden attributed to the PMM2-CDG manifestations has also been recently reported^12^.

Despite research efforts, a clear correlation between genotype and phenotype is not known. However, certain early clinical markers may indicate greater neurological involvement, such as the coexistence of lipodystrophy and inverted nipples^10^, and while some studies suggest that more severe cerebellar atrophy may be associated with greater cognitive impairment^16,17^. Nevertheless, whether molecular characteristics, laboratory studies, or clinical manifestations are connected to adaptive and functional characteristics, as well as caregiver stress, remains unexplored.

In the present study we aimed to: (1) assess the adaptive behavior profile and conduct a psychopathological screening of pediatric and adult PMM2-CDG patients in two cohorts, (2) evaluate the impact of the disease on parental stress, and (3) explore correlations between the study results and clinical characteristics of patients.

## Methods

This cross-sectional study recruited individuals with a confirmed molecular diagnosis of PMM2-CDG via the CDG Spanish Consortium from May to October 2022. Patients whose parents did not sign the informed consent or lacked a confirmed molecular diagnosis were excluded. The manuscript has been prepared in accordance with the STROBE statement.

Epidemiological and molecular data, laboratory and neuroimaging findings, including cerebellum midsagittal vermis relative diameter (MVRD)^16^, dysmorphology traits^10^, and personal antecedents, were collected.

Adaptive functioning data were collected by mental health professionals (SMPS and ML) from parents and adults who completed the tests during their regular visits (16/21 children, 8/16 adults) or via telephone (5/21 children, 8/16 adults). In all the cases parents were the main caregivers. Statistical association of adaptive behavioral scale results with clinical features (epilepsy, stroke-like episodes, vascular events, coagulation abnormalities, transaminases abnormalities, thyroid function) and dysmorphological traits was analyzed.

### Measures

Cerebellar syndrome was assessed using the International Cooperative Ataxia Rating Scale (ICARS)^16,18^, which employs a 100-point rating scale with a higher score denoting more evident clinical abnormalities. Global disease severity was evaluated with the Nijmegen Pediatric CDG Rating Scale (NPCRS)^19^, organized around three domains: current function (section I), system-specific involvement (section II), and current clinical assessment (section III). Based on the scores, patients were classified as mild (0–14), moderate (15–25), or severe (≥ 26).

Behavioral and adaptive evaluation, psychopathological screening, and parental stress evaluation were made with several tests, all validated in Spanish. Some tests were common to the whole sample (Vineland Adaptive Behavior Scale, Third edition (VABS-3)^20^, and the Parental Stress Index-Short form, Fourth edition (PSI-SF)^21^, and some were specific to age (Health of the Nation Outcome Scales for Children and Adolescents (HoNOSCA)^22^ and the Child Behavior Checklist (CBCL), for patients below 18 years of age^23^, the Nation Outcome Scales for People with Learning Disabilities (HoNOS-LD)^24,25^, and the Symptom Check-List-90R (SCL-90R)^26^, for adult patients) (Supplementary material).

1. Adaptive behavior and functioning

The VABS-3 is a caregiver-completed assessment that measures adaptive behavior. It yields standard scores in different domains: communication, daily living skills, and socialization. These domain scores are combined to generate an overall Adaptive Behavior Composite (ABC) score. Higher scores indicate better adaptive functioning, while lower scores suggest challenges in adaptive behavior. Additionally, equivalent developmental age can be calculated. Since the VABS3 is designed to measure adaptive behavior of individuals from birth to age 90, for analytical purposes, three age groups were defined ad hoc: children (6 years of age to under 12 years of age), adolescents (12 years of age to under 18 years of age), and adults (over 18 years of age). They were evaluated separately in three severity groups defined in this case as severely affected if their ABC score was below the median value of the sample and age group. Conversely, individuals were categorized as mildly affected if their ABC score was above that median value.

The whole group was evaluated using the HoNOSCA for children and the HoNOS-LD for adults, as measures of general health and social functioning^22,24,25^.

2. Psychopathological screening

The CBCL is a widely used caregiver report for evaluating behavioral, emotional, and social problems in children and adolescents. The scale sets provide scores associated with disorders from the Diagnostic and Statistical Manual of Mental Disorders^27^, including affective problems, anxiety problems, somatic problems, attention deficit with hyperactivity (ADHD), oppositional defiant problems and conduct problems, demonstrating good reliability when compared to psychological diagnoses^28^.

To screen adults for a range of psychopathological features, the SCL-90R, a multidimensional questionnaire for people with learning disabilities and mental health needs, was applied^26^.

3. Parental stress

PSI-SF is a short test designed to evaluate the magnitude of stress in the parent–child system^21^. It includes self-report questionnaire about three major domains: Parental Distress, Parent–Child Dysfunctional Interaction, and Difficult Child, which combines to form a Total Stress scale. After obtaining the direct scores, a percentile score can be obtained. Scores 85th percentile or higher are considered clinically significant.

### Data analysis

Descriptive statistics were calculated for numerical variables, and frequency tables were generated for categorical variables. Normality in numerical variables was assessed with QQ plots. Differences in numerical variables between two groups were studies with Student's t test or Mann–Whitney's U test, and ANOVA or Kruskall-Wallis tests were used in comparisons between more than 2 groups. Tukey's test or pairwise Mann–Whitney's U test were performed as post-hoc analysis for ANOVA and Kruskall-Wallis tests, respectively. Effect sizes were calculated for these tests using Cohen’s d (for Student’s t test), rank-biserial correlation (r) (for Mann–Whitney’s U test), η^2^ (for ANOVA) and η^2^(H) (for Kruskal–Wallis test). The relationship between categorical variables was studied with Chi-square test or Fisher’s exact test. Pearson and Spearman’s correlations were used to study the relationship between numerical variables. Correlation coefficients above 0.7 were considered strong correlations, while values between 0.5 and 0.7 are considered moderate correlations. All statistical tests were two-sided. *P* values < 0.05 were considered significant. Statistical analysis was carried out using SPSS V.23.0 (Armonk, NY: IBM Corp.).

### Ethical issues

All procedures followed were in accordance with the ethical standards of the responsible committee on human experimentation (institutional and national) and with the Helsinki Declaration of 1975, as revised in 2000. Ethical permission for the study (Internal Approval Code: PIC-108-14) was obtained from the Research & Ethics Committee of the SJD Research Foundation (https://www.irsjd.org/en/research/ceim/). Parents gave their written informed consent, and children/adolescents and adults gave their assent. For children below 16 years of age, informed consent was obtained from all subjects and/or their parents/legal guardian. Adult patients gave their written informed consent when possible. Samples were obtained in accordance with the Helsinki Declaration of 1964, as revised in October 2013 in Fortaleza, Brazil.

## Results

Thirty-seven individuals affected by PMM2-CDG were evaluated. Twenty-one were children (mean age 12.4 years; standard deviation (SD) 3.8; twelve males, nine females), and 16 were adults (mean age 29.3 years; SD 8.0; nine males, seven females). Individuals’ characteristics and previous diagnoses in the neurodevelopmental area are presented in Table 1. For mental health monitoring, in children, three out of 21 had regular psychologist visits, and two visited both psychologists and psychiatrists. In adults, two out of 16 had regular psychiatrist follow-ups, and another one visited a psychologist regularly.

**Table 1:** Cohort description: epidemiological, laboratory and clinical description.

Patients	Children (N = 21)	Adults (N = 16)
Sex (male/female)	12/9	9/7
Age (mean, SD [range])	12.4 years, 3.8 [4.4–17.6]	29.3 years, 8.0 [18.1–44.6]
Laboratory studies		
Abnormal liver function	8/20 (40%)	1/11 (9%)
Coagulation		
Abnormally low ATIII values	7/18 (38.8%)	9/11 (81.8%)
Abnormally low FIX values	0/18 (0.0%)	0/5 (0.0%)
Abnormally low FXI values	6/18 (33.3%)	2/5 (40.0%)
Abnormal thyroid function	1/19 (5.2%)	0/9 (0.0%)
Sialotransferrin profile (mean percentage, SD [range])		
Tetrasialotransferrin	60.4, 13.2 [35.2–80.2]	56.7, 19.2 [32.3–84.8]
Disialotransferrin	21.9, 12.0 [2.7–46.7]	20.1, 12.8 [1.0–29.0]
Asialotranferrin	4.6, 4.4 [0.0–14.6]	6.1, 5.5 [0.0–12.7]
Sialotransferrin ratios (mean ratio, SD [range])		
Mono/dioligo ratio	0.35, 0.28 [0.03–0.61]	0.32, 0.24 [0.01–0.61]
A/dioligo ratio	0.08, 0.09 [0.00–0.27]	0.10, 0.09 [0.00–0.23]
Vascular events	3/21 (14.2%)	2/16 (12.5%)
Dysmorphic traits		
Typical facial gestalt	17/21 (80.9%)	16/16 (100.0%)
Lipodystrophy	17/21 (80.9%)	12/16 (75.0%)
Inverted nipples	5/21 (23.8%)	11/16 (68.7%)
Long lender fingers	6/21 (28.4%)	14/16 (87.5%)
Neurodevelopmental and general assessment		
Epilepsy	8/21 (38.1%)	4/16 (25.0%)
Stroke like events	8/21 (38.1%)	6/16 (37.5%)
Developmental delay	21/21 (100.0%)	16/16 (100.0%)
Intellectual disability	20/21 (95.2%)	14/16 (87.5%)
ADHD	12/21 (57.1%)	NCD
Autistic spectrum disorder	6/21 (28.6%)	NCD
Obsessive–compulsive disorder	2/21 (14.3%)	NCD
ICARS (mean, SD [range])	43.4, 25.2 [14–76]	38.2, 17.7 [4–58]
NPCRS (mean, SD [range])	18.3, 7.5 [6–28]	17.5, 4.5 [6–22]
MVRD (mean, SD [range])	43.2, 16.2 [35–56]	45.4, 25.9, [30–59]
Walking abilities	9 Sev, 5 Mod, 7 Mild	6 Sev, 3 Mod, 7 Mild
Speech	8 Sev, 4 Mod, 9 Mild	4 Sev, 5 Mod, 7 Mild
School/occupational activities	13 Sev, 3 Mod, 5 Mild	6 Sev, 6 Mod, 4 Mild
Molecular findings (variants present at least in two alleles)*		
p.R141H (16/74 alleles, 21.6%), p.T237M (7/74 alleles, 9.5%), p.V44A (4/74 alleles, 5.4%), p.R123Q, p.P113L, and p.F207S (3/74 alleles, 4.1%), p.L32R, p. D65Y, p. F157S, and p.G214S (2/74 alleles, 2.7%)		

Walking abilities: Severe (Sev) no autonomous walk at all; Moderate (Mod) walk with help (person/device); Mild (Mild) independent walk.

Speech and comunication: Severe (Sev) no funcional speech; Moderate (Mod) verbal intention, difficult to understand; Mild (Mild) communication mainly using speech.

School/occupational activities: Severe (Sev) special school or occupational centre; Moderate (Mod) regular school with major adaptation; Mild (Mild) regular with little adaptation.

*SD* Standard Deviation; range is in square brackets; *ADHD* Attention deficit and hyperactive disorder, *NCD* No complete data.

*All the subjects have biallelic variants in PMM2, with only two exceptions that showed homozygous variants.

The mean NPCRS value for children was 18.3 (SD 7.5), corresponding to a moderate categorization as a group. In fact, nine of them were classified as moderate. For adults, the mean NPCRS value was 17.5 (SD 4.5), with eleven of them categorized as moderate. Out of 21 children, eight were unable to complete the ICARS scale due to comprehension difficulties. Thirteen children were evaluated, with a mean ICARS score of 43.4 over 100 (SD 25.2). In the adult group, three individuals were unable to perform the ICARS maneuvers, and two could not be evaluated. Eleven ICARS assessments were conducted, resulting in a mean score of 38.2 over 100 (SD 17.7).

### Adaptive behavior and functioning

**Figure 1:**
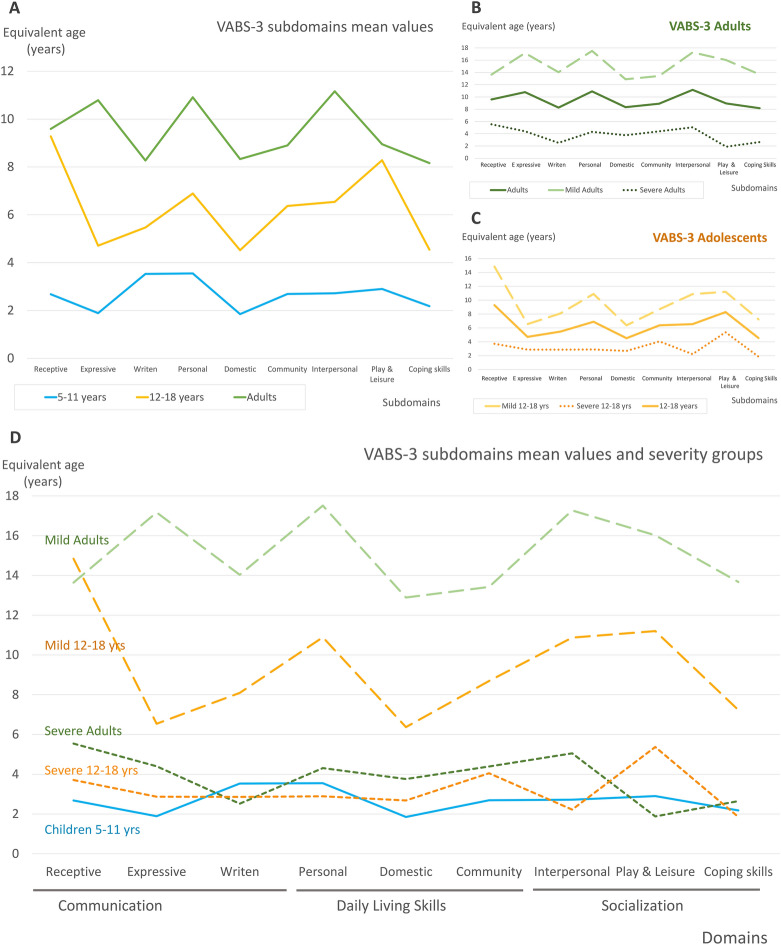
VABS-3 equivalent developmental ages and domains. The equivalent developmental age for three age groups: Adults (green), adolescents (12–18 years, yellow), and children (5–11 years, blue). Patients under 5 have been excluded to facilitate homogeneity. (**A**) Shows the mean values of equivalent developmental age for every age group in the different subdomains. The graph shows an increase in equivalent developmental age across all domains. The subdomain ‘Domestic’ (domain ‘Daily living skills’) is severely affected in all age groups not reaching nine years of equivalent developmental age. This subdomain indicates the individual's abilities and independence in performing everyday tasks according to the individual’s age and related to household activities and routines (personal hygiene, meal preparation, cleaning, managing personal belongings, etc.). The other severely affected subdomain is ‘Coping skills’ (domain ‘Socialization’), also consistently affected across the ages and related to an individual's ability to demonstrate behavior and emotional control in different situations involving others. (**B**) and (**C**) show the equivalent developmental age in different subdomains for adolescents and adults including the mean scores (continuous line), the mean scores of the severely affected (those with ABC score below the median value, scores represented by the dotted lines) and the mean scores of the mildly affected (those with ABC score above the median value, scores represented by the dashed lines). (**D**) Further illustrates the equivalent developmental age for severely affected adolescents and adults (represented by the dotted lines), mildly affected adults and adolescents (represented by the dashed lines) and the group of children. Both severely affected adolescents and adults present equivalent ages that are lower than or close to five years of age. In contrast, the mildly affected groups show some improvement in the subdomains as they age, although they still do not reach the expected age level.

With regard to VABS-3, there were no statistically significant differences between sex and age groups, even when considering the split of the sample of children into young children and adolescents. VABS-3 results of young children (n = 7), adolescents (n = 12) and adults (n = 16) are presented in Fig. 1. As domains, ‘Daily living skills’ was the most severely affected specific domain. For both age groups all domains and the ABC score were affected (all mean values were in the low range) (Table 2).

**Table 2:** Domains and subdomains of the VABS-3: means, interquartile ranges and ranges with age equivalents.

Children	Standard score			Equivalent age (yrs)		
Domain	Mean (SD)	Median (IQR)	Range	Mean (SD)	Subdomains	Correlation with NPCRS sections*
Communication	62.5 (27.9)	68.0 (43.5)	20.0–115.0	7.86 (7.88)	Receptive	SI: r = − 0.831, *p* = 0.000 SIII: r = − 0.756, *p* = 0.000
				4.15 (4.73)	Expressive	
				4.82 (3.74)	Writing	
Daily living skills	57.9 (22.5)	58.0 (36.0)	20.0–114.0	6.32 (6.59)	Personal	SI: r = − 0.640, *p* = 0.003 SIII: r = − 0.582, *p* = 0.009
				4.23 (4.25)	Domestic	
				5.40 (3.40)	Community	
Socialization	69.3 (25.0)	73.0 (47.5)	31.0–122.0	6.15 (7.34)	Interpersonal relationships	
				6.89 (6.46)	Play and leisure	
				4.61 (5.42)	Coping skills	
ABC score	68.5 (20.1)	69.0 (18.0)	29.0–121.0			SI: r = − 0.737, *p* = 0.000 SIII: r = − 0.575, *p* = 0.01

Adults	Standard score			Equivalent age (yrs))		
Domain	Mean (SD)	Median (IQR)	Range	Mean (SD)	Subdomains	Correlation with NPCRS sections*
Communication	60.0 (30.9)	60.0 (66.3)	20.0–100.0	9.59 (8.56)	Receptive	SI: r = − 0.671, *p* = 0.017
				10.79 (9.53)	Expressive	
				8.27 (7.33)	Writing	
Daily living skills	52.3 (28.6)	49.0 (58.5)	20.0–92.0	10.91 (9.38)	Personal	SI: r = − 0.734, *p* = 0.007
				8.33 (7.46)	Domestic	
				8.90 (5.88)	community	
Socialization	63.9 (33.0)	67.0 (69.3)	20.0–107.0	11.16 (9.38)	Interpersonal relationships	
				8.95 (9.26)	Play and leisure	
				8.16 (7.72)	Coping skills	
ABC score	63.9 (28.9)	73.5 (58.5)	20.0–100.0	9.59 (8.56)		SI: r = − 0.621, *p* = 0.031

Total	Standard score					
Domain	Mean (SD)	Median (IQR)	Range			
Communication	61.5 (28.8)	68.0 (51.5)	20.0–115.0			
Daily living skills	55.5 (25.1)	57.0 (45.0)	20.0–114.0			
Socialization	67.0 (28.4)	73.0 (48.5)	20.0–122.0			
ABC score	66.5 (24.0)	69.0 (29.0)	20.0–121.0			

Vineland descriptors: high (scores of 130–140), moderately high (scores of 115–129), adequate (scores of 86–114), moderately low (scores of 71–85) and low (scores of 20–70); yrs: years.

*SD* Standard deviation; *IQR* Interquartile range; *ABC* Adaptive behavior composite; *SI* Section I of NPCRS; *SIII* Section III of NPCRS.

*Using Spearman test, only significant moderate or strong correlations with the NPCRS sections are shown: SI for section I, SII for section II, and SIII for section III.

Considering subdomains, ‘Domestic’ in the domain ‘Daily living skills’, and ‘Coping skills’ in the ‘Socialization’ domain, were the most affected across the ages (Fig. 1A). There was a general improvement with age in all the domains. Conversely, when the evaluation was performed clustering patients into age and severity groups, the most severely affected adolescents and adult patients maintained an adaptive functionality level under that for an equivalent age of 6 years (Fig. 1B,C and D).

**Figure 2:**
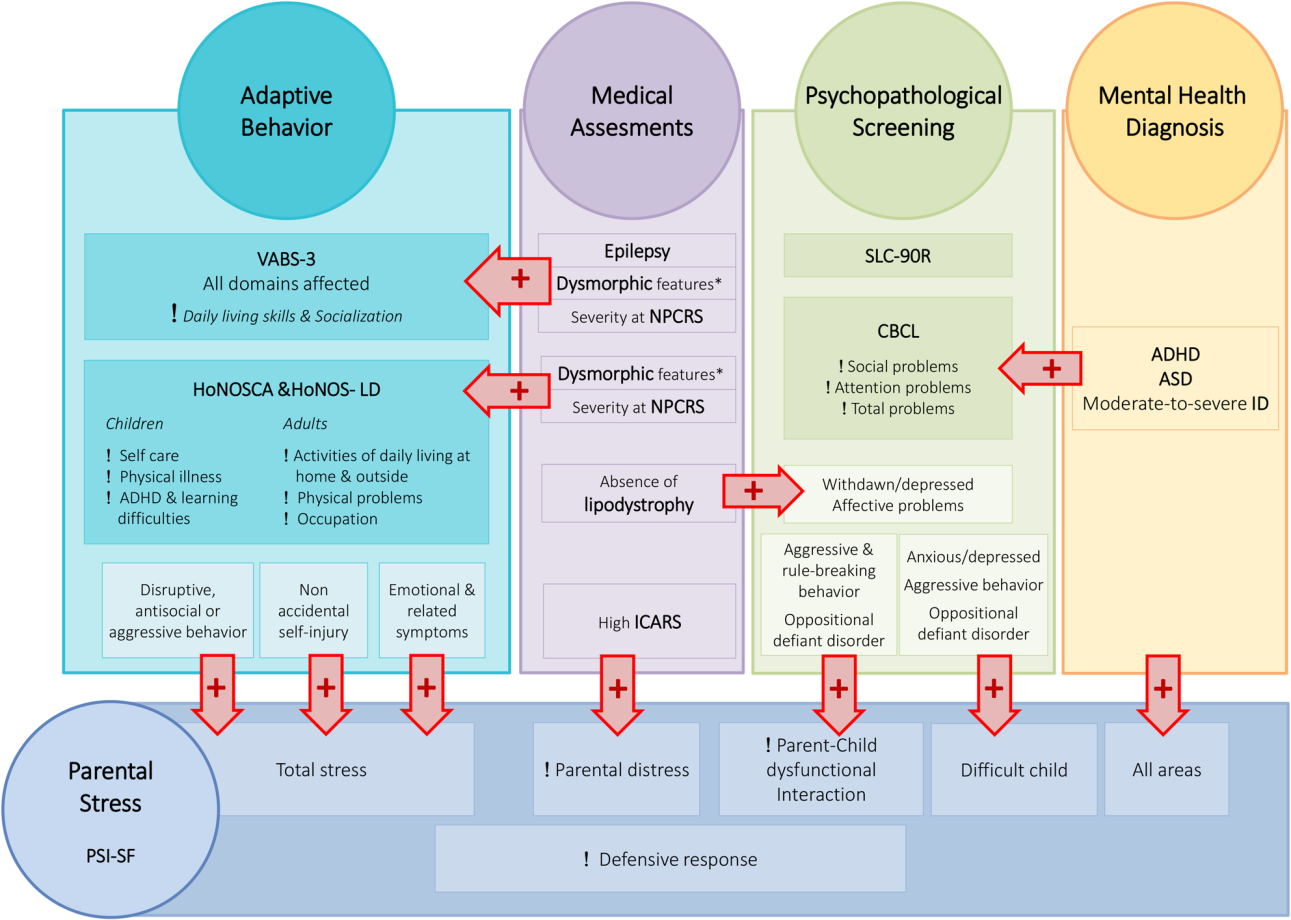
Assessments results and their interrelations. The altered areas or domains are highlighted using an exclamation sign. Statistically significant relations between the assessments are marked using red arrows. *Dysmorphic features evaluated were typical facial gestalt, lipodystrophy and inverted nipples. Student T test showed lower scores in different VABS-3 areas: communication skills (mean 70.2 vs. 43.2, *p* = 0.006), daily living skills (63.0 vs. 39.8, *p* = 0.007), and ABC score (75.1 vs. 48.7, *p* = 0.001). Individuals with inverted nipples showed lower scores in all the domains: ‘Communication’ (83.8 vs. 54.0, *p* = 0.004), ‘Daily living’ (75.6 vs. 48.4, *p* = 0.002), ‘Socialization’ (88.1 vs. 60.2, *p* = 0.006), and ‘ABC score’ (82.7 vs. 59.9, *p* = 0.009). Lipodystrophy associated lower scores in the four domains: ‘Communication’ (mean 88.4 vs. 54.8, *p* = 0.002), ‘Daily living’ (80.6 vs. 48.9, *p* = 0.001), ‘Socialization’ (88.9 vs. 62.0, *p* = 0.015), and ‘ABC score’ (86.0 vs. 60.6, *p* = 0.007). Regarding HoNOS tests, characteristic gestalt, inverted nipples, and lipodystrophy were, each statistically associated with pathological scores in ‘Physical illness’, and ‘Self-care’ item, both in children and adults. ANOVA test showed a significant relation between NPCRS severity categories and abnormal scores in VABS-3 domains: ‘Communication’, ‘Daily living’, ‘Socialization’, and ‘ABC score’. Chi square test showed statistically significant associations between NPCRS and the altered areas in the HoNOS tests. The absence of lipodystrophy was related to an increase in significant scores for ‘Withdrawn/depressed’ (*p* = 0.04) and also ‘Affective problems’ (*p* = 0.035) in the CBCL evaluation. There was a positive Spearman correlation between the higher ICARS results and the highest Parental Distress domain scores (r: 0.648, *p* = 0.031). Higher scores in the total stress scale on the PSI correlated with higher scores in four items on the HoNOSCA: ‘Disruptive, antisocial or aggressive behavior’ (r: 0.615, *p* = 0.003), ‘Non accidental self-injury’ (r: 0.624, *p* = 0.002), and ‘Emotional and related symptoms’ (r: 0.738, *p* = 0.000). There was a statistically positive association between significant scores in the Parent–Child Dysfunctional Interaction item and significant scores at the CBCL (‘Aggressive and rule-breaking behavior’ and ‘Oppositional and defiant disorder’) and a correlation between scores in ‘Difficult child’ and ‘Total stress’ and some CBCL scores, showing a Pearson’s *r* greater than 0.7 in all cases.

Lower scores in three different domains were associated with the presence of epilepsy (‘Communication skills’, ‘Daily living skills’, and ‘ABC score’), and individuals with inverted nipples showed lower scores in all the domains. Also, lipodystrophy was related to lower scores in the four domains (Fig. 2). There were no other statistically significant associations with laboratory findings or medical features, including cerebellar measure.

There was a relation between NPCRS severity categories and abnormal scores in all VABS-3 domains (Communication domain *p* = 0.002, η^2^ = 0.34; Daily living skills domain *p* = 0.001, η^2^ = 0.37; Socialization domain *p* = 0.03, η^2^ = 0.19; and ABC score *p* = 0.004, η^2^ = 0.31). No correlation between VABS-3 domain results and raw scores in total NPCRS was found. However, scores in sections I and III of the NPCRS showed negative significant correlations with ‘Communication’, ‘Daily living skills’ and ‘ABC score’ domains in children. Total score in section I also shed negative significant correlation with ‘Communication’, ‘Daily living skills’ and ‘ABC score’ domains in adults (Table 2). For ICARS, no statistical correlations were found with the VABS-3 domain results, but those patients unable to complete an ICARS evaluation due to intellectual disability presented significantly lower mean scores in all VABS-3 domains.

Concerning the HoNOSCA results, there were no differences among sex groupings. HoNOSCA identified moderate-to-severe problems in more than 50% of the children in the following areas: ‘Scholastic or language skills’ (95.2%), ‘Physical illness or disability problems’ (76.2%), ‘Self-care’ (76.2%), ‘Non-organic somatic symptoms’ (symptoms that do not have an identifiable organic or medical cause, where psychological or emotional factors may be underlying) (66.7%), and ‘Overactivity attention and concentration’ (52.4%) (Supplementary Table 1).

There were statistically significant associations between severity measured with NPCRS and HoNOSCA in the areas with identified problems (Supplementary Table 1). The presence of dysmorphic traits such as characteristic gestalt, inverted nipples, and lipodystrophy were each statistically associated with pathological scores in the HoNOSCA scale in different items (Fig. 2, Supplementary Table 1). There was no relation between significant scores and the listed laboratory findings or medical problems.

Regarding the results of HoNO’S-LD for adults, it also identified moderate-to-severe problems in more than 50% of the patients in the following areas, in order of prevalence: ‘Activities of daily living at home’ and ‘outside the home’ (81.3%), ‘Physical problems’ (62.5%), and ‘Occupation and activities’ (56.3%). There were no differences between the sexes. The higher severity seen through the NPCRS was associated with higher results in the ‘Activities of daily living at home’ and ‘outside the home’. As with children, the presence of inverted nipples and lipodystrophy was statistically associated with pathological scores in different HoNO’S-LD items in the adult group (Supplementary Table 2).

### Psychopathological screening

The assessment of behavioral emotional and social problems using the CBCL showed that ‘Social problems’, ‘Internalizing problems’ and ‘Attention problems’ were the most significant, including around 50% of individuals in Limit or Significant values (Table 3). The ‘Total Problems’ score also yielded more than 50% of the subjects with scores in the limit or significance range, denoting a high level of overall behavioral and emotional difficulties.

**Table 3:** Child behavior checklist (CBCL) assessment results.

CBCL-children					
Syndrome scale	Mean (SD)	Median (IQR)	Range	Limit (%)	Significant (%)
(1) Anxious/depressed*	54.7 (6.7)	51.0 (8.0)	50.0–70.0	3/21 (14.3)	1/21 (4.8)
(2) Withdrawn/depressed	57.0 (6.8)	57.0 (12.0)	50.0–70.0	2/21 (9.5)	2/21 (9.5)
(3) Somatic complaints	59.9 (7.8)	57.0 (11.0)	50.0–78.0	4/21 (19.0)	0/21 (0.0)
(4) Social problems*	65.2 (8.2)	67.0 (8.5)	50.0–85.0	**8/21 (38.1)**	**5/21 (23.8%)**
(5) Thought problems*	59.5 (7.9)	57.5 (12.0)	50.0–76.0	3/21 (15.0)	2/21 (9.5)
(6) Attention problems*	62.9 (10.5)	59.0 (15.0)	51.0–87.0	**2/21 (9.5)**	**5/21 (23.8)**
(7) Rule-breaking behavior*	53.5 (5.4)	51.0 (4.0)	50.0–70.0	0/21 (0.0)	1/21 (4.8)
(8) Aggressive behavior*	61.0 (12.4)	57.0 (13.5)	50.0–92.0	1/21 (4.8)	4/21 (19.0)
Internalizing*	56.0 (9.0)	58.0 (14.0)	39.0–70.0	**5/21 (23.8)**	**4/21 (19.0)**
Externalizing*	56.0 (10.4)	54.0 (17.0)	40.0–78.0	0/21 (0.0)	**6/21 (28.6)**
Total problems*	60.2 (9.0)	60.0 (13.0)	45.0–78.0	**4/21 (19.0)**	**7/21 (33.3)**
Affective problems*	59.6 (7.3)	60.0 (11.0)	50.0–75.0	**5/21 (23.8)**	**2/21 (9.5)**
Anxiety problems*	60.0 (7.9)	60.0 (13.0)	50.0–73.0	0/21 (0.0)	5/21 (23.8)
Somatic problems	57.9 (8.6)	56.0 (13.5)	50.5–77.0	1/21 (4.8)	3/21 (15.0)
Attention deficit disorder*	59.8 (9.2)	57.0 (10.5)	50.0–77.0	**3/21 (25.0)**	**4/21 (19.0)**
Oppositional defiant disorder*	59.7 (10.1)	58.0 (7.5)	50.0–80.0	2/21 (9.5)	4/21 (19.0)
Conduct disorder*	54.0 (7.6)	50.0 (4.5)	50.0–77.0	2/21 (9.5)	1/21 (4.8)

Problems showing limit or significant scores in more than 30% of the sample are in bold.

*SD* Standard deviation; *IQR* Interquartile range.

*Higher scores in these items were statistically associated with higher scores in PSI in the following domains: Parental distress, Parent–child dysfunctional interaction, difficult child, total stress.

Regarding sex, there were no differences, with the only exception being ‘Anxiety problems’, which were more prevalent in males (*p* = 0.045, Cohen’s d = 0.95). The absence of lipodystrophy was associated with an increase in significant scores for ‘Withdrawn/depressed’ and also ‘Affective problems’. There was no other statistically significant relation with other dysmorphic traits, laboratory findings, clinical evaluations or medical problems.

On evaluating the psychological symptoms and psychopathological features of adults using the SCL-90R, no differences between the sexes were found. No relevant comorbidities were evident, with only the Positive Distress item significant in four out of 16 patients (Supplementary Table 3).

### Parental stress

Regarding the magnitude of stress assessed through the PSI-SF, a high prevalence of clinically significant results (near 50% or greater) was found, both in the ‘Parental Distress’ items focused on parental feelings of distress, such as anxiety, depression, and feeling overwhelmed, and also those ‘Parent–Child Dysfunctional Interaction’ items exploring issues like discipline problems, attachment difficulties, and disrupted communication in the parent–child relationship. There were no statistical differences between age or sex groups. Although not significant, ‘Defensive response’ and ‘Parental distress’ showed the highest results in children (Table 4). High scores were found in the ‘Defensive response’ domain, which evaluates whether parents are being completely forthcoming in their responses. Parents of five children and three adults had high or clinically significant results in all the items of PSI-SF, meaning a score above the 85th percentile. All of those patients presented a formal diagnosis of mental health disorder such as ADHD, autism spectrum disorder (ASD) and/or, moderate-to-severe intellectual disability (Fig. 2).

**Table 4:** Parenting stress index, fourth edition short form.

Domain and age groups	Percentile mean (SD)		Parents showing significant scores*	Percentage of parents with significant scores* (%)
Defensive response	Children	71.4 (30.3)	12/20	60.0
	Adults	60.5 (34.3)	4/16	25.0
	Total	68.2 (32.3)	16/37	43.2
Parental distress	Children	65.1 (32.3)	11/21	52.4
	Adults	43.4 (29.9)	3/16	18.8
	Total	55.7 (32.7)	14/37	37.8
Parent–child dysfunctional interaction	Children	64.3 (34.3)	10/21	47.6
	Adults	55.6 (29.7)	5/16	31.3
	Total	60.5 (32.2)	15/37	40.5
Difficult child	Children	54.7 (36.1)	7/21	33.3
	Adults	42.0 (35.3)	3/16	18.8
	Total	49.2 (35.8)	10/37	27.0
Total stress scale	Children	64.1 (34.2)	10/21	47.6
	Adults	45.2 (36.0)	4/16	25.0
	Total	55.9 (35.8)	14/37	37.8

*SD* Standard deviation; *IQR* Interquartile range.

*Concerning parents’ answers, 85th percentile and above are considered clinically significant scores.

There were no statistically significant associations between the patients’ severity measured with the NPCRS, laboratory findings, clinical characteristics or the VABS-3 assessment and results in PSI-SF scores. In contrast, there was a positive correlation between the higher ICARS results and the higher Parental Distress domain scores. There was a statistically positive association between significant scores in several PSI-SF items and the significant scores on the CBCL and the HoNOSCA (Fig. 2, Table 3 and 4).

## Discussion

PMM2-CDG is the best-known type of CDG. However, this is the first study to evaluate the adaptive behavior, functioning and psychopathological profile of PMM2-CDG patients, something of significant concern for families. Intellectual difficulties, problems with daily activities and self-direction, along with behavioral disturbances, limit patients' autonomy and negatively impact the quality of life of both patients and their families^12^. However, the assessment of these clinical behavioral and cognitive characteristics is not typically included in physician evaluations or clinical trial protocols, and no tools have been specifically tested in this patient group.

Although, developmental delay is common in PMM2-CDG patients, intellectual disability is not always present^4,10^. Regarding learning disabilities, HoNOSCA results indicated that scholastic difficulties were present in almost all the patients. In the child group, more than 50% received a formal ADHD diagnosis, although we believe that comprehensive evaluation of executive functions was not conducted for the entire sample, particularly for those in the adult group. Additionally, the presence of an intellectual disability can hinder formal diagnosis of ADHD, even though it is often observed in genetically determined neurodevelopmental conditions^29^. ASD is also very prevalent in this cohort, having been diagnosed in almost one third of patients. Nevertheless, the presence of ASD traits (lack of cognitive flexibility, difficulties in socialization, and lack of skills in communication apart from speech) not fulfilling all the ASD criteria and not leading to a formal diagnosis, but still deserving of attention, is probably greater, as is the case in other neurogenetic conditions^29^. Importantly, the cerebellum has been identified as playing a significant role in both executive and emotional functions, potentially contributing to the features observed in ADHD and ASD^7–9^.

In our PMM2-CDG sample, no significant correlations were found between cerebellar atrophy and adaptive functioning or psychopathological findings in contrast with a previous report^30^. As a major limitation, it should be noted that the neuroimaging studies were evaluated retrospectively; they were obtained at very different ages, and, it is likely that as significant atrophy becomes more pronounced with age and residual volume is affected, the ability to differentiate between phenotype severities with imaging may diminish^16^.

VABS-3 has been widely used to describe the adaptive phenotype of neurodevelopmental conditions and inborn errors of metabolism, and also as a tool to evaluate the efficacy of interventions^31,32^. Our study shows that all the domain scores and their equivalent developmental ages were low, for both age groups. The domain ‘Daily Living Skills’ was the most severely affected, indicating difficulties in performing age-appropriate everyday tasks, including personal, domestic, and community activities. The next most affected domain was ‘Communication’. Communication difficulties are a frequent source of distress for both parents and patients, often leading to frustration associated with challenges in expression and comprehension.

When evaluating by subdomains, two were consistently lower than the others across the sample: ‘Domestic’ (household task performance) and ‘Coping skills’ (how the individual demonstrates behavior and emotional control in different situations involving others), directly impacting autonomy and functionality. Although our study design is not longitudinal, it shows general improvement across the different age groups in all the domains. But evaluating mildly and severely affected patients as a different subset, it is seen that, across the different age groups of severely affected patients, there is little adaptive skills improvement; they maintained an adaptive functionality under an equivalent age of six years. Conversely, in mildly affected patients, adaptive skills exhibit improvement across age groups. Additional research employing a longitudinal design is imperative to substantiate the observed trend of stabilization or improvement with age, particularly within a subset of patients.

The presence of epilepsy, the categorization of the NPCRS as severe, and higher values in section I of NPCRS, but also section III in the case of children, were associated with lower scores in VABS-3. Also, higher NPCRS categorization was associated with pathological scores in different areas of HoNOS scales, denoting a consistency among the different evaluations.

Inverted nipples and lipodystrophy were highlighted as severity signs in a previous study^10^. The group of subjects presenting them showed statistically significant lower scores in almost all the domains and subdomains of VABS-3 (Fig. 2), denoting difficulties in adaptive behavior, but also abnormal scores in different HoNOS scales items (Supplementary Tables 1 and 2), again highlighting their significance as markers of severity. Together with epilepsy, they may indicate a more severely affected subset of patients in terms of adaptive functioning. The association between clinical severity, adaptive functioning limitations, and dysmorphic traits in PMM2-CDG is likely grounded in the underlying mutations. These mutations might impact both physical features and the overall clinical presentation, establishing the connection between the early dysmorphic traits and the global severity of the condition. Today, given the absence of a known genotype–phenotype correlation, the presence of lipodystrophy and inverted nipples, both detected early in the newborn or infant, probably points towards greater adapting functioning limitations and, latterly, a more severe neurological phenotype.

In summary, VABS-3 and HoNOS scales point up the difficulties in daily living activities, particularly domestic tasks, as well as coping skills, but also in scholastic, occupational and working tasks, alerting to a severe impact on patients’ autonomy and social relationships caused by the disease.

CBCL results showed significant social difficulties and attention problems, affecting around 50% of individuals. These difficulties appeared to be related to communication and executive functions, as indicated by the VABS-3 assessment. Unfortunately, this study lacks a formal assessment of executive functions, despite their significance as an abnormal domain in both CBCL and VABS-3.

Surprisingly, anxiety problems were found to be more prevalent in boys, which is in contrast to the general population^33^, what is described in intellectual disability groups^34^, and to a recently reported PMM2-CDG series^30^, all showing more risk of developing anxiety in the female group. Emotional problems and depression were not found to be relevant problems by any of the screening tools. Another recent study, by Ligezka et al.^35^, collecting patient-reported outcomes, did not find differences on anxiety and depression domains in PMM2-CDG patients compared to the general population, but the analysis of PROMIS domain T-scores by age showed higher anxiety and depression scores in patients older than 18 years compared to younger patients, and the analysis by gender showed higher anxiety and depression symptoms in women. Remarkably, a subset of patients with absence of lipodystrophy showed a statistically significant correlation to an increase in the existence of significant ‘Withdrawn/depressed’ and also ‘Affective problems’. In an attempt to explain this apparently incongruent fact, if lipodystrophy has been previously described as a severity indicator^10^, patients who do not present it may be more conscious of their difficulties and therefore it has a greater impact on their emotional situation and self-esteem.

PSI-SF evaluates stress in the parent–child system, which is expected to some extent. A notable finding was the high scores in the ‘Defensive response’ domain, which evaluates whether parents are being completely forthcoming in their responses. This high score denotes the tendency to minimize or normalize the real impact of stress in their lives. The ‘Parental distress’ domain, reflecting difficulties in adjusting to parenthood, showed the highest scores, particularly in children, highlighting the importance of finding ways to reduce distress through community and social resources. Interestingly, higher ICARS scores, as a sign of motor cerebellar syndrome, correlated with higher scores in the ‘Parental distress’ domain. Behavioral disturbances identified by the CBCL are associated with more pathological scores on most items, and the presence of comorbidities such as ADHD, ASD, and moderate-to-severe intellectual disability is also relevant, as all individuals with clinically significant results in all PSI-SF items have one of these neurodevelopmental disorders. In conclusion, both motor impairments and behavioral complexity contribute to increased burden and distress among caregivers. Importantly, parenting interventions may ameliorate the family burden but also favor the parent–child interaction.

## Conclusions

Our study highlights different areas of adaptive deficits that need to be prioritized in future intervention programs and therapies. ‘Daily living skills’ was the most severely affected domain, both in the VABS-3 and HoNOS tests, directly impacting autonomy. ‘Communication’ domain was also clearly affected. Evaluation of self-sufficiency and functionality, which may alter school activities and occupational tasks but also leisure, deserve attention as has been overlooked in clinical papers on PMM2-CDG.

Our study introduces validated tools available in different languages and widely recognized in the field for assessing and monitoring adaptive functioning. We suggest the use of VABS-3 in the regular patients’ evaluation since it offers a comprehensive assessment of adaptive functioning and daily living skills, crucial domains affected in PMM2-CDG.The study shows external correlation of the tests with clinical indicators of severity, lending support to their use in the clinical practice. These tools also include a standardized assessment of the family's stress burden. Considering their high impact on autonomy, adaptive functioning, psychopathological risks, and family stress should be taken into account in evaluation, monitoring, and intervention programs for PMM2-CDG patients, including therapies under development.

### Supplementary Information

Supplementary Information 1.

Supplementary Information 2.

## Data Availability

The datasets used and/or analysed during the current study are available from the corresponding author on reasonable request.

## References

[1] Freeze HH, Chong JX, Amshad MJ, Ng BG (2014). Solving glycosylation disorders: fundamental approaches reveal complicated pathways. Am. J. Hum. Genet..

[2] Grunewald S, Matthijs G, Jaeken J (2002). Congenital disorders of glycosylation: A review. Pediatr. Res..

[3] Schiff M, Roda C, Monin ML (2017). Clinical, laboratory and molecular findings and long-term follow-up data in 96 French patients with PMM2-CDG (phosphomannomutase 2-congenital disorder of glycosylation) and review of the literature. J. Med. Genet..

[4] Vals MA, Morava E, Teeäär K (2017). Three families with mild PMM2-CDG and normal cognitive development. Am. J. Med. Genet. A.

[5] Antoun H, Villeneuve N, Gelot A, Panisset S, Adamsbaum C (1999). Cerebellar atrophy: An important feature of carbohydrate deficient glycoprotein syndrome type 1. Pediatr. Radiol..

[6] Aronica E, van Kempen AA, van der Heide M (2005). Congenital disorder of glycosylation type Ia: A clinicopathological report of a newborn infant with cerebellar pathology. Acta Neuropathol..

[7] Adamaszek M, D'Agata F, Ferrucci R (2017). Consensus paper: Cerebellum and emotion. Cerebellum.

[8] Schmahmann JD (1998). Dysmetria of thought: Clinical consequences of cerebellar dysfunction on cognition and affect. Trends Cogn. Sci..

[9] Tedesco AM, Chiricozzi FR, Clausi S, Lupo M, Molinari M, Leggio MG (2011). The cerebellar cognitive profile. Brain.

[10] Martinez-Monseny A, Cuadras D, Bolasell M (2019). From gestalt to gene: Early predictive dysmorphic features of PMM2-CDG. J. Med. Genet..

[11] Gámez A, Serrano M, Gallego D, Vilas A, Pérez B (2020). New and potential strategies for the treatment of PMM2-CDG. Biochim. Biophys. Acta Gen. Subj..

[12] Pascoal C, Ferreira I, Teixeira C (2022). Patient reported outcomes for phosphomannomutase 2 congenital disorder of glycosylation (PMM2-CDG): Listening to what matters for the patients and health professionals. Orphanet J. Rare Dis..

[13] Patton KA, Ware R, McPherson L (2018). Parent-related stress of male and female carers of adolescents with intellectual disabilities and carers of children within the general population: A cross-sectional comparison. J. Appl. Res. Intellect. Disabil..

[14] Minnes P, Perry A, Weiss JA (2015). Predictors of distress and well-being in parents of young children with developmental delays and disabilities: The importance of parent perceptions. J. Intellect. Disabil. Res..

[15] Fitzgerald J, Gallagher L (2021). Parental stress and adjustment in the context of rare genetic syndromes: A scoping review. J. Intellect. Disabil..

[16] Serrano NL, De Diego V, Cuadras D (2017). A quantitative assessment of the evolution of cerebellar syndrome in children with phosphomannomutase-deficiency (PMM2-CDG). Orphanet J. Rare Dis..

[17] Serrano M, de Diego V, Muchart J (2015). Phosphomannomutase deficiency (PMM2-CDG): Ataxia and cerebellar assessment. Orphanet J. Rare Dis..

[18] Trouillas P, Takayanagi T, Hallett M (1997). International cooperative ataxia rating scale for pharmacological assessment of the cerebellar syndrome. The ataxia neuropharmacology committee of the world federation of neurology. J. Neurol. Sci..

[19] Achouitar S, Mohamed M, Gardeitchik T (2011). Nijmegen paediatric CDG rating scale: A novel tool to assess disease progression. J. Inherit. Metab. Dis..

[20] Sparrow S. S., Cicchetti D. V. & Saulnier C. A. Vineland Adaptive Behavior Scales, Third Edition (Vineland-3) (Pearson, 2016).

[21] Abidin R (2012). Parenting Stress Index Fourth Edition (PSI-4).

[22] Gowers SG, Harrington RC, Whitton A (1999). Brief scale for measuring the outcomes of emotional and behavioural disorders in children. Health of the nation outcome scales for children and adolescents (HoNOSCA). Br. J. Psychiatry.

[23] Achenbach T, Edelbrock C (1991). The Child Behavior Checklist Manual.

[24] Ashaye O, Mathew G, Dhadphale M (1997). A comparison of older longstay psychiatric and learning disability inpatients using the health of the nation outcome scales. Int. J. Geriatr. Psychiatry.

[25] Esteba-Castillo S, Torrents-Rodas D, García-Alba J, Ribas-Vidal N, Novell-Alsina R (2018). Translation and validation of the Spanish version of the health of the nation outcome scales for people with learning disabilities (HoNOS-LD). Rev. Psiquiatr. Salud Ment. (Engl Ed).

[26] Peveler RC, Fairburn CG (1990). Measurement of neurotic symptoms by self-report questionnaire: Validity of the SCL-90R. Psychol. Med..

[27] American Psychiatric Association (2013). Diagnostic and Statistical Manual of Mental Disorders.

[28] Warnick EM, Bracken MB, Kasl S (2008). Screening efficiency of the child behavior checklist and strengths and difficulties questionnaire: A systematic review. Child Adolesc. Ment. Health.

[29] Waite J, Heald M, Wilde L, Woodcock K, Welham A, Adams D, Oliver C (2014). The importance of understanding the behavioural phenotypes of genetic syndromes associated with intellectual disability. Paediatr. Child Health.

[30] Pettinato F, Mostile G, Battini R (2021). Clinical and radiological correlates of activities of daily living in cerebellar atrophy caused by PMM2 mutations (PMM2-CDG). Cerebellum.

[31] Pearson E, Watkins A, Oliver C, Karim A, Clayton-Smith J, Welham A (2021). The adaptive functioning profile of Pitt-Hopkins syndrome. Eur. J. Med. Genet..

[32] Ahmed A, Rudser K, King KE (2022). Quantifying medical manifestations in Hurler syndrome with the infant physical symptom score: Associations with long-term physical and adaptive outcomes. Mol. Genet. Metab..

[33] Kovess-Masfety V, Woodward MJ, Keyes K (2021). Gender, the gender gap, and their interaction; analysis of relationships with children's mental health problems. Soc. Psychiatry Psychiatr. Epidemiol..

[34] Sáez-Suanes GP, García-Villamisar D, Pozo Armentia AD (2023). Does the gender matter?: Anxiety symptoms and emotion dysregulation in adults with autism and intellectual disabilities. Autism Res..

[35] Ligezka AN, Mohamed A, Pascoal C (2022). Patient-reported outcomes and quality of life in PMM2-CDG. Mol. Genet. Metab..

