# Supplementary material for: Untangling adaptive functioning of PMM2-CDG across age and its impact on parental stress: a cross-sectional study
Source: Sci Rep. 2023 Dec 20;13:22783. doi: 10.1038/s41598-023-49518-y (PMC10739927; doi:10.1038/s41598-023-49518-y)
Supplement: Supplementary file 1 — Supplementary Information 1. [file 41598_2023_49518_MOESM1_ESM.docx]

**Supplementary material**

*Measures and Assessments*

The Vineland Adaptive Behavioral Scale, third edition (VABS-3) is an individually administered measure of adaptive behavior [1]. The Parent/Caregiver form was used, which was completed by a parent or caregiver who is knowledgeable about the every-day functioning of the subject. Parents describe the examinee's adaptive behavior using a rating scale format.

The VABS-3 computes standard scores across domains which are then summed to generate an overall Adaptive Behavior Composite (ABC), based on scores for three specific adaptive behavior domains:

- The Communication domain measures describe how well the individual listens and understands, expresses himself through speech, and reads and writes.
- The Daily Living Skills domain assesses the individual's performance of the practical, everyday tasks of living that are appropriate for his age.
- The individual's score for the Socialization domain reflects his functioning in social situations.

Item responses are collected on a 3-point Likert scale with values representing 0 (never), 1 (sometimes), and 2 (usually or often) to capture the frequency of each target behavior. The domain scores are also expressed as standard scores with a mean of 100 and standard deviation of 15. An optional Motor Skills (gross and fine) domain score was not administered this scale due to the bias due to intrinsic motor impairments associated with PMM2-CDG. Vineland provide suggested qualitative descriptors of high (scores of 130–140), moderately high (scores of 115–129), adequate (scores of 86–114), moderately low (scores of 71–85) and low (scores of 20–70).

PSI-4-SF Parenting Stress Index™, Fourth Edition Short Form is an abbreviated version of the full-length PSI-4 [2], designed to evaluate the magnitude of stress in the parent–child system. It includes 36 items focuses on three major domains of stress: Parental Distress (made up of the first 12 items), Parent-Child Dysfunctional Interaction (items 13 to 24), and Difficult Child (items 25 to 36), which combine to form a Total Stress scale. Items 37 to 55 provide qualitative information but are not taken into account in the correction. All items (1 to 36) are evaluated on a scale of 1 to 5 being: Strongly Agree: Score 5, Agree: Score 4, Don't know: Score 3, Disagree: Score 2 and Strongly Disagree: Score 1 (except items 22,32 and 33, with reverse score). The score for each of the factors would be the sum of the scores for the items that make up the factor. After obtaining the direct or raw scores, a percentile score on the recording sheet can be obtained. The normal range of score is with in the 16th to 84th percentiles. Scores 85th percentile or higher are clinically significant.

PSI-4-SF Parenting Stress Index™, Fourth Edition Short Form, also includes a Defensive Response scale. This scale assesses the extent to which the examinee approaches the questionnaire with a greater or lesser bias to present the most favorable self-image and to minimize indications of stress problems in the parent-child relationship. To obtain this score, the values of the responses to items 1, 2, 3, 7, 8, 9 and 11 are added together. Extremely low scores, raw score of 10 or less, on the defensive response scale suggest the parent is trying to provide the image of a highly competent, the parent is not invested in the role and therefore is not experiencing the usual stresses associated with caring for the child, or is in fact a very competent person who handles the responsibilities of parenting very well.

The Child Behavior Checklist (CBCL) is a widely used caregiver report to assess behavioral emotional and social problems in children and adolescents (ages 6 to 18), often used as a diagnostic screen [3]. The CBCL's questions are associated with problems on a syndrome scale in eight different categories: (1) anxious/depressed, (2) withdrawn/depressed, (3) somatic complaints, (4) social problems, (5) thought problems, (6) attention problems, (7) rule-breaking behavior, and (8) aggressive behavior. Some of these scales are grouped into internalizing (anxious/depressed, withdrawn/depressed, somatic complaints) and externalizing (rule-breaking behavior, aggressive behavior) problems. The CBCL also has a scale set to show scores associated with disorders from the Diagnostic and Statistical Manual of Mental Disorders (anxiety, oppositional defiant disorder, conduct problems, somatic problems, affective problems, and attention deficit disorder) [4]. Many studies have demonstrated a high rate of reliability between the scales of the CBCL and actual psychological diagnosis [5]. According to the normative data of the CBCL, a t-score ≤ 59 indicates non-clinical symptoms, a t-score between 60 and 64 indicates that the child is at risk for problem behaviors, and a t-score ≥ 65 indicates clinical symptoms.

The whole group of individuals (children, adolescents and adults) were evaluated using the HoNOSCA (Health of the Nation Outcome Scales for Children and Adolescents) and the HoNO’S-LD (Health of the Nation Outcome Scales for People with Learning Disabilities and Mental Needs) [6,7]. HoNOSCA is a measure of outcome for use in child and adolescent mental health services focusing on general health and social functioning. The measure is a 15 item questionnaire indicating the severity of each problem, on a scale of 0-4. The measure is made up of two sections. The first section consists of 13 items relating to different types of problems: (1) Disruptive, antisocial or aggressive behavior, (2) Overactivity attention and concentration, (3) Non accidental self-injury, (4) Alcohol, substance/solvent misuse, (5) Scholastic or language skills, (6) Physical illness or disability problems, (7) Hallucinations and delusions, (8) Non-organic somatic symptoms, (9) Emotional and related symptoms, (10) Peer relationships, (11) Self-care and independence, (12) Family life and relationships and (13) Poor school attendance. The second section consists of two items relating the parent or young person’s knowledge of the nature of the young person’s difficulties and their information about the services available.

HoNOS-LD is an instrument for measuring outcome in people with learning disabilities with additional mental health needs. It consists of 18 items relating to different types of problems: (1) Behavioral problems directed at others, (2) Behavioral problems directed at self (self-aggression), (3) Other mental and behavioral problems, (4) Attention and concentration, (5) Memory and orientation, (6) Communication (understanding), (7) Communication (expression), (8) Problems associated with hallucinations or delusions, (9) Problems associated with mood swings, (10) Sleep disorders, (11) Problems with eating and drinking, (12) Physical problems, (13) Epilepsy, (14) Activities of daily living at home, (15) Activities of daily living outside the home, (16) Self-care, (17) Relational problems, and (18) Occupation and activities.

For both scales, each item is rated on a 5-point scale of severity (0, no problem, to 4, meaning severe to very severe problem), scores of 2-to-4 on any item should indicate the need of intervention.

The SCL-90R is a multidimensional questionnaire developed to screen for a range of psychological symptoms and psychopathological features for people with Learning disabilities and Mental Health Needs [8]. It is evaluated and interpreted in terms of nine primary dimensions: (1) Somatizations, (2) Obsessions and compulsions, (3) Interpersonal sensitivity, (4) Depression, (5) Anxiety, (6) Hostility, (7) Phobic anxiety, (8) Paranoid ideation, and (9) Psychoticism; and three global indexes of psychological distress: (1) Global severity index, (2) Positive Distress Index and (3) Total positive symptoms. Each of the 90 items is answered on a five-point scale (0-4).It is evaluated and interpreted in terms of nine primary dimensions and three global indices of psychological distress. A T score of 65 or higher is considered indicative of a person at risk. Any score equal to or higher than T 80 indicates the presence of severe pathology.

**References**

1. Sparrow S. S., Cicchetti D. V., Saulnier C.A. Vineland Adaptive Behavior Scales, Third Edition (Vineland-3). TX: Pearson. San Antonio. 2016
2. Abidin, R. Parenting Stress Index Fourth Edition (PSI-4); Psychological Assessment Resources: Odessa. FL. USA. 2012

Achenbach T, Edelbrock C. The child behavior checklist manual. Burlington: The University of Vermont; 1991

1. American Psychiatric Association. Diagnostic and Statistical Manual of Mental Disorders. 5th ed. Arlington, VA: American Psychiatric Association. 2013

Warnick EM, Bracken MB, Kasl S. Screening Efficiency of the Child Behavior Checklist and Strengths and Difficulties Questionnaire: A Systematic Review. Child Adolesc Ment Health. 2008;13:140-7.

Gowers SG, Harrington RC, Whitton A, et al. Brief scale for measuring the outcomes of emotional and behavioural disorders in children. Health of the Nation Outcome Scales for children and Adolescents (HoNOSCA). Br J Psychiatry. 1999;174:413-6.

Esteba-Castillo S, Torrents-Rodas D, García-Alba J, Ribas-Vidal N, Novell-Alsina R. Translation and validation of the Spanish version of the Health of the Nation Outcome Scales for People with Learning Disabilities (HoNOS-LD). Traducción y validación de la versión española de la escala Health of the Nation Outcome Scales for People with Learning Disabilities (HoNOS-LD). Rev Psiquiatr Salud Ment (Engl Ed). 2018;11:141-50.

Peveler RC, Fairburn CG. Measurement of neurotic symptoms by self-report questionnaire: validity of the SCL-90R. Psychol Med. 1990;20:873-9.
