# Supplementary material for: Untangling adaptive functioning of PMM2-CDG across age and its impact on parental stress: a cross-sectional study
Source: Sci Rep. 2023 Dec 20;13:22783. doi: 10.1038/s41598-023-49518-y (PMC10739927; doi:10.1038/s41598-023-49518-y)
Supplement: Supplementary file 2 — Supplementary Information 2. [file 41598_2023_49518_MOESM2_ESM.docx]

Supplementary Table 1. HoNOSCA results for children.

| **HoNOSCA - Children**  **Types of problems** | **Children with significant score/total (percentage)** | **Statistically Significant Associations***  **(P value, size effect)** |
| --- | --- | --- |
| (1) Disruptive, antisocial or aggressive behavior | 5/21 (23.8%) |  |
| (2) Overactivity attention & concentration | 11/21 (52.4%) |  |
| (3) Non accidental self-injury | 2/21 (9.5%) |  |
| (4) Alcohol, substance/solvent misuse | 0/21 (0%) |  |
| (5) Scholastic or language skills | 20/21 (95.2%) | Higher results: NPCRS severity category (p=0.026, η^2^(H)=0.16) |
| (6) Physical illness or disability problems | 16/21 (76.2%) | Higher results in:  Typical gestalt (p=0.013, r=0.86)  Lipodystrophy (p=0.013, r=0.92)  Inverted nipples(p=0.001, r=0.99)  NPCRS severity category (p=0.001, η^2^(H)=0.39 ) |
| (7) Hallucinations and delusions | 0/21 (0%) |  |
| (8) Non-organic somatic symptoms | 14/21 (66.7%) | Higher results in:  Inverted nipples (p=0.014, r=0.77)  NPCRS severity category (p=0.007, η^2^(H)=0.23) |
| (9) Emotional and related symptoms | 3/21 (14.2%) |  |
| (10) Peer relationships | 6/21 (28.6%) |  |
| (11) Self-care and independence | 16/21 (76.2%) | Higher results in:  Typical gestalt (p=0.001, r=0.83)  Lipodystrophy (p=0.032, r=0.83)  Inverted nipples (p=0.005, r=0.89)  NPCRS severity category (p=0.001, η^2^(H)=0.37) |
| (12) Family life and relationships | 4/21 (19%) |  |
| (13) Poor school attendance | 1/21 (4.8%) |  |

* Associations were evaluated using Chi square test and Kruskal-Wallis test. P value and size effect is shown.

Scholastic or language skills, Physical illness or disability problems, Self-care, Non-organic somatic symptoms, and Overactivity attention & concentration are remarkable in the children group. There were statistically significant associations between severity measured by NPCRS, the presence of dysmorphic traits such as characteristic gestalt, inverted nipples, and lipodystrophy, but not with other listed medical problems.

Supplementary Table 2. HoNOS LD results for adults.

| **HoNOS LD - Adults**  **Types of problems** | **Subjects with significant score/total (percentage)** |
| --- | --- |
| (1) Behavioral problems directed at others | 2/16 (12.5%) |
| (2) Behavioral problems directed at self (self-aggression) | 0/16 (0%) |
| (3) Other mental and behavioral | 3/16 (18.8%) |
| (4) Attention and concentration | 6/16 (37.5%) |
| (5) Memory and orientation | 3/16 (18.8%) |
| (6) Communication (understanding) | 3/16 (18.8%) |
| (7) Communication (expression) | 5/16 (31.3%) |
| (8) Problems associated with hallucinations or delusions | 0/16 (0%) |
| (9) Problems associated with mood swings | 1/16 (6.3%) |
| (10) Sleep disorders | 1/16 (6.3%) |
| (11) Problems with eating and drinking | 0/16 (0%) |
| (12) Physical problems | 10/16 (62.5%) |
| (13) Epilepsy | 1/16 (6.3%) |
| (14) Activities of daily living at home | 13/16 (81.3%)* |
| (15) Activities of daily living outside the home | 13/16 (81.3%)* |
| (16) Self-care | 6/16 (37.5%) |
| (17) Relational problems | 2/16 (12.5%) |
| (18) Occupation and activities | 9/16 (56.3%) |

* Chi square test showed statistically significant association with NPCRS severity category (p=0.038, r=0.96 and p=0.038, r=0.96, respectively)

HoNO’S-LD for adults, also identified moderate-to-severe problems in the following areas: Activities of daily living at home and outside the home, Physical problems, and Occupation and activities. The higher severity seen through the NPCRS was associated with higher results in the Activities of daily living at home and outside the home. As with children, the presence of inverted nipples and lipodystrophy was statistically associated with pathological scores different items.

Supplementary Table 3. SCL-90R results: Evaluation of the psychological symptoms and psychopathological features of adults with PMM2-CDG.

| **SCL-90R Adults** | | | | | |
| --- | --- | --- | --- | --- | --- |
|  | Mean (SD) | Median (IQR) | Range | Limit Score (%) | Significant Score (%) |
| **Global indexes of psychological distress:** | | | | | |
| (1) Global severity index | 53.9 (6.6) | 51.5 (3.0) | 50.0-76.0 | 0/16 (0.0) | 1/16 (6.3) |
| (2) Positive Distress | 64.6 (8.7) | 63.5 (11.5) | 51.0-83.0 | 2/16 (12.5) | 4/16 (25.0) |
| (3) Total positive symptoms | 51.4 (1.3) | 51.5 (2.0) | 50.0-54.0 | 0/16 (0.0) | 0/16 (0.0) |
| **Primary dimensions:** | | | | | |
| (1) Somatizations | 55.1 (7.0) | 52.5 (8.0) | 50.0-76.0 | 0/16 (0.0) | 1/16 (6.3) |
| (2) Obsessions and compulsions | 53.8 (4.2) | 52.5 (4.8) | 50.0-64.0 | 0/16 (0.0) | 0/16 (0.0) |
| (3) Interpersonal sensitivity | 51.6 (1.7) | 51.0 (3.0) | 50.0-54.0 | 0/16 (0.0) | 0/16 (0.0) |
| (4) Depression | 53.8 (4.4) | 52.0 (4.0) | 50.0-65.0 | 1/16 (6.3) | 0/16 (0.0) |
| (5) Anxiety | 54.1 (4.9) | 52.5 (7.0) | 50.0-66.0 | 1/16 (6.3) | 0/16 (0.0) |
| (6) Hostility | 51.9 (3.4) | 50.0 (2.0) | 50.0-63.0 | 0/16 (0.0) | 0/16 (0.0) |
| (7) Phobic anxiety | 51.5 (3.5) | 50.0 (2.0) | 50.0-64.0 | 0/16 (0.0) | 0/16 (0.0) |
| (8) Paranoid ideation | 51.5 (3.0) | 50.0 (1.8) | 50.0-60.0 | 0/16 (0.0) | 0/16 (0.0) |
| (9) Psychoticism | 51.5 (3.3) | 50.0 (1.8) | 50.0-63.0 | 0/16 (0.0) | 0/16 (0.0) |

SD: standard deviation; IQR: interquartile range.

SCL-90R results showed no relevant comorbidities, with only the Positive Distress item being significant in four out of 16 patients
